# Beauty Is Not Always a Perk: The Role of Attractiveness and Social Interest in Trust Decisions

**DOI:** 10.3390/bs15020175

**Published:** 2025-02-07

**Authors:** Junchen Shang, Yizhuo Zhang

**Affiliations:** Department of Medical Humanities, School of Humanities, Southeast University, Nanjing 211189, China; 220224090@seu.edu.cn

**Keywords:** beauty premium, social interest, trust decisions, facial attractiveness, vocal attractiveness

## Abstract

This study examined the impact of males’ facial and vocal attractiveness, as well as social interest in females’ decision-making in a trust game. The results showed that trustees with attractive faces or expressing positive social interest were more likely to receive initial investments. Trustees with attractive voices also received more initial investments than unattractive ones in most conditions, except when they had attractive faces and positive interest. Moreover, participants reinvest in trustees with attractive faces or voices, even if they withheld repayment. However, trustees with positive interest would receive more reinvestment only when they reciprocated. In addition, trusters expressing positive social interest were expected to invest and earn repayment at higher rates. Nonetheless, trusters with attractive faces (or voices) were only expected to invest at higher rates when they had attractive voices (or faces) and negative interest. These findings suggest that beauty premium is modulated by participants’ roles, such that the effect of beauty would be stronger when participants encounter trustees rather than trusters. Positive social interest is a perk in most conditions, except when trustees withheld repayment.

## 1. Introduction

Trust is widely recognized in the literature as a key benefit linked to attractiveness (Stirrat & Perrett, 2010; Póvoa et al., 2020). Although our parents have taught us not to judge a book by its cover from a young age, why do we still instinctively place trust in those who are attractive? Ample research proposes that people tend to attribute a body of positive traits—competence, honesty, integrity, credibility, etc.—to attractive individuals (Patzer, 1983; Wilson & Eckel, 2006; Maestripieri et al., 2017). Social psychologists explain this as the idea that attractiveness activates a stereotype, which influences perceptions and judgments of people in a relatively direct way (Wilson & Eckel, 2006). This stereotype can further evolve into the halo effect, supporting the assumption that attractive people may always perform well (Thorndike, 1920). Therefore, the first impression created by attractiveness can affect how others perceive a person as a whole, causing them to judge an attractive person as more credible. Most research about attractiveness and trust focused on facial attractiveness and vocal attractiveness. What makes a face attractive or unattractive? Previous studies have shown that critical characteristics, such as averageness, symmetry, and sexual dimorphism in the face, influence facial attractiveness (Baudouin & Tiberghien, 2004; Fink et al., 2006; Rhodes, 2006). Faces having average facial configurations are considered more attractive. Completely symmetric faces are also rated as more attractive. Female faces that have more feminine features are attractive, while the influence of masculinity on male facial attractiveness remains less clear. Likewise, what makes a voice attractive or unattractive? Acoustic feature parameters such as pitch, frequency, and formant are efficient predictors of vocal attractiveness (Zhang et al., 2020). For example, women rate male voices, which have lower fundamental frequencies (pitch), lower formant frequencies, and smaller formant dispersion, as more attractive, while men prefer female voices with opposite acoustic features (Zheng et al., 2020; Shang & Zhang, 2024b).

Many researchers have employed the trust game paradigm to investigate the relationship between trust and attractiveness (FeldmanHall et al., 2018; Li et al., 2017). In this game, the trusters own a certain amount of investment capital, and they decide whether to invest a portion of the capital in another partner, known as the trustees (Chen et al., 2012). It should be noted that investment behaviors in the game carry some risk. If the truster invests the money, the trustee will receive a multiplied reward, typically three or four times the original investment. Then the trustee can choose to return half of the reward to the truster, making them earn more than their original investment. But if the trustee opts to keep all rewards for themselves, the truster will not receive any return, resulting in a loss. As a result, the investment behavior of the truster and the reciprocation behavior of the trustee are regarded as indicators of trust decision-making (Wilson & Eckel, 2006).

There are prosocial biases in favor of attractive-faced adults in trust decisions. Attractive faces trigger the “beauty premium” effect, where trusters are inclined to invest more money in these attractive trustees (Chen et al., 2012). For instance, Voit et al. (2021) found that participants entrusted more money to partners with attractive faces. Póvoa et al. (2020) discovered that female trustees received higher investments when wearing makeup compared to when they were makeup-free, particularly from male trusters, who invested more money in makeup for female trustees than their female counterparts did. Chen et al. (2012), using ERP technology, further investigated the psychological underpinnings underlying facial attractiveness in trust decisions. They discovered that attractive faces elicited smaller P2 and larger feedback-related negativity (FRN) amplitudes compared to unattractive ones. These results suggest that the discrimination of facial attractiveness occurs rapidly and automatically during the early processing stage. And participants were more inclined to expect attractive partners to reciprocate their investments, which led to a larger FRN amplitude difference, highlighting the influence of facial attractiveness on trust-related expectations. However, Wilson and Eckel (2006) found the “beauty penalty” in trust decisions when participants assumed the role of trustees, such that participants had higher expectations for attractive trusters, and if attractive trusters did not invest as much as participants’ expectations, the participants would withhold reciprocation, even repaying less money than they would to unattractive trusters. In other words, when counterparts failed to meet participants’ expectations, participants would show beauty penalty in their responses. Also, some researchers suggested that the beauty premium was tempered by participants’ roles in the ultimatum game (Ma et al., 2022); for male participants, female recipients elicited a greater beauty premium than female proposers. Therefore, the effect of facial attractiveness on trust decisions may vary depending on the roles involved.

Similar to facial attractiveness, vocal attractiveness induced beauty premium in the trust game where participants acted as trusters. Shang and Liu (2022b) found that participants entrusted more to partners with attractive voices. Attractive voices induced larger P3 and more positive late positive complex (LPC) than unattractive voices, while only male voices elicited larger N1 amplitudes in attractive voice conditions. These findings not only indicate gender differences in trust decisions but also suggest that attractive voices may be more effective at capturing early attention and evoking positive emotional experiences. Subsequently, Shang and Liu (2022a) further demonstrated a difference in the FRN amplitudes between attractive and unattractive voices: the rewarding effect of attractive voices weakened the negative impact of economic losses, promoting trust investments. These studies suggest that the psychological underpinnings behind the premium effect of vocal attractiveness is similar to that of facial attractiveness in trust decisions. Recently, Shang and Zhang (2024b) investigated the influence of audiovisual-integration attractiveness and social interest in the ultimatum game and dictator game, where participants assumed various roles. When participants acted as proposers, they allocated more money to those counterparts with attractive faces or voices; however, as recipients, the attractiveness effects of visual and auditory both disappeared. In addition to beauty premium, they found that counterparts who expressed positive social interest, which conveys positive and friendly social signals through language (Jones et al., 2008), also earned monetary preference. Nonetheless, how the effects of facial attractiveness, vocal attractiveness, and social interest existing simultaneously on trust decision-making with switching gaming roles remains unclear.

Additionally, in Shang and Zhang’s (2024b) one-shot games, participants never received feedback between decisions and could not adjust expectations in subsequent moves. It is unclear whether beauty and positive social interest guarantee better treatment when working in concert with feedback (gain or loss) in trust decisions. Shang and Zhang’s (2024b) study used female participants and male stimuli, so it remains unknown whether gender differences exist. Our key research question focused on the influence of facial and vocal attractiveness and social interest on trust decisions involving different roles and feedback. Due to complex experimental design and availability concerns, like Shang and Zhang’s (2024b) study, we continue to use female participants and male stimuli.

To investigate these questions, we used a modified trust game (TG) (Wilson & Eckel, 2006; Shang & Liu, 2022b), where participants played as both trusters and trustees with counterparts who varied in facial and vocal attractiveness and social interest. Especially, we explored decisions before and after receiving feedback (gain or loss) from counterparts, respectively. We proposed the following hypotheses. Firstly, when acting as trusters (TG1), as in previous research (Chen et al., 2012; Shang & Liu, 2022a), participants would invest more in trustees with attractive faces or voices before they received feedback from trustees. Afterwards, if attractive trustees withheld repayment (negative feedback), since people have higher expectations of attractive counterparts (Wilson & Eckel, 2006), participants’ reinvestments may decrease. Secondly, when acting as trustees (TG2), participants would also have higher expectations of attractive trusters before they knew the information of investment (feedback from trusters) since attractive people were expected to send more (Wilson & Eckel, 2006). Then, if they received investment, participants would be more willing to reciprocate attractive trusters (Shang & Zhang, 2024b). The impact of positive social interest may be similar to that of attractiveness (Yuan et al., 2024). Additionally, building on Shang and Zhang’s (2024b) research, the first movers (proposers who had the right to divide money) may consider more information than the second movers (recipients). Likewise, in the trust game, we anticipated a three-way interaction between social interest, facial attractiveness, and vocal attractiveness when participants acted as trusters, especially the effect of attractiveness may be modulated by social interest.

## 2. Material and Methods

We used More*Power 6.0.4 software (Campbell & Thompson, 2012) to calculate a sample size of 52, with an effect size (*η_p_*^2^) of 0.14 and a statistical test power of 0.80 (*α* = 0.05) (Shang & Zhang, 2024b). Totally, 62 female students from non-economics and non-psychology majors in Southeast University were recruited. Data of two participants were excluded due to misunderstanding of experimental rules, two were excluded due to accidental interference, and one was excluded for expressing doubts about the authenticity of the task, finally leaving 57 valid sets of data (*M*_age_ = 23.53 years, *SD* = 2.52). All participants signed informed consent and received appropriate compensation. All research was performed in accordance with the Declaration of Helsinki and approved by the Ethics Committee of the Psychology Research Center at Southeast University.

A 2 (facial attractiveness: attractive, unattractive) × 2 (vocal attractiveness: attractive, unattractive) × 2 (social interest: positive, negative) within-participant design was conducted, where the ratio of initial investment/reinvestment (TG1) and the ratio of expected investment/reciprocation (TG2) were dependent variables.

A total of 16 male faces (half were attractive) were adapted from Shang and Zhang (2024a, 2024b). The independent samples *t*-test demonstrated a significant difference in the nine-point attractiveness ratings (1 = “very unattractive”, 9 = “very attractive”, same with the voices) between attractive faces (*M* = 6.31, *SD* = 0.42) and unattractive faces (*M* = 3.11, *SD* = 0.46), *t*(14) = 15.23, *p* < 0.001, 95% CI = [2.34, 3.06]. A total of 16 male voices (4 attractive voices with positive interest, 4 attractive voices with negative interest, 4 unattractive voices with positive interest, and 4 unattractive voices with negative interest) were also adapted from Shang and Zhang (2024a, 2024b). These voices were recorded in advance from another group of male participants in Mandarin Chinese. Positive and negative interests were conveyed via voice stimuli. The positive social interest voice read “我喜欢你”, which means “I like you”, while the negative social interest voice read “我不喜欢你”, which means “I don’t like you”. Two-way ANOVA with attractiveness and social interest as independent factors was conducted on the vocal attractiveness rating differences. There was a significant difference between attractive voices (*M* = 6.47, *SD* = 0.35) and unattractive voices (*M* = 3.92, *SD* = 0.24), *F*(1, 12) = 215.55, *p* < 0.001, *η_p_*^2^  = 0.89. Neither the main effect of social interest nor the interaction between attractiveness and social interest was significant, *F*s ≤ 0.24, *p*s ≥ 0.629. These results indicated that the levels of stimuli were distinct and effective. Both attractive and unattractive faces were divided into 4 groups: One-fourth of attractive faces were paired with attractive voices of positive social interest. One-fourth of attractive faces were paired with attractive voices of negative social interest. One-fourth of attractive faces were paired with unattractive voices of positive interest. One-fourth of attractive faces were paired with unattractive voices of negative interest. The grouping of unattractive faces was also performed similarly. Each face was solely paired with one voice.

Before the experiment, we informed participants that all partners in the game were real people whose faces, voices, and decisions had been collected in advance by researchers (Shang & Zhang, 2024b; Shang & Liu, 2022b). The order of TG1 (participants acted as trusters) and TG2 (participants acted as trustees) was counterbalanced between participants. In the game, trusters owned CNY 20 at the beginning of the experiment and would decide whether to invest CNY 0.5 in a trustee in each trial. If the trustee received the investment, the investment was quadrupled by the researcher, and the trustee would decide whether to send reciprocation (half of the increased sum) back to the truster or to keep the money, leaving the truster with nothing. Participants were told that their income could be accumulated by the investment and reciprocation.

**Figure 1:**
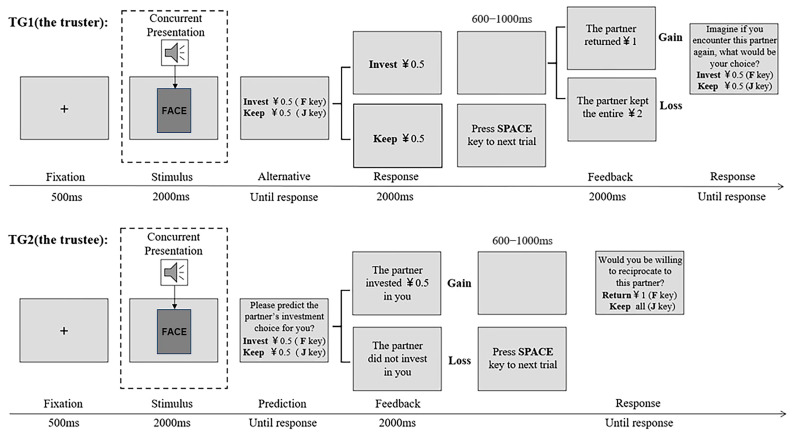
Schematic representation of the trust game.

The experimental procedure is as follows (see Figure 1): A central fixation was presented for 500 ms, followed by a face–voice pair of the partner appearing for 2000 ms. Then, in TG1, two sentences, “invest ¥0.5” and “keep ¥0.5”, appeared on the screen, and participants were given unlimited time to decide whether to invest in trustees. Once participants submitted their choices, the decision would be presented for 2000 ms to emphasize the outcome. If participants decided to invest, after a blank screen lasting 600–1000 ms, the feedback (“The partner returned ¥1” or “The partner kept the entire ¥2”) was displayed for 2000 ms. Finally, participants were asked to make the no-time-limit reinvestment choice, imagining whether they would invest when they had a chance to interact with the same trustee in the future. If participants refused to invest, their money would not change. At the end of each trial, participants were informed to press the space key to continue. In TG2, after the 500 ms fixation, a face–voice pair of the partner appeared for 2000 ms, and participants were given unlimited time to predict whether the truster would invest in them. Once their predictions were made, the feedback (“The partner invested ¥0.5 in you” or “The partner did not invest in you”) was displayed for 2000 ms. If participants received investment, after a blank screen lasting 600–1000 ms, they would decide whether to reciprocate to the partner (“return ¥1” or “keep all”). At the end of each trial, participants pressed the space key to continue. The response keys (“F/J”) were counterbalanced between participants. Each task consisted of 16 practice trials and 64 experimental trials. Each combination of face and voice was presented four times (half with gain trials) in each task in a pseudo-random manner, where the same combination of stimuli cannot appear consecutively twice within a block.

For the manipulation check of stimuli selection, after the experiment, participants rated the attractiveness of faces and voices separately on a nine-point scale (1 = “very unattractive”, 9 = “very attractive”). In addition, we asked all participants the following: “Do you believe that it is a real game?” This question involves two aspects: first, whether participants believe the game partners are real persons, and second, whether they believe that investment and return decisions in the game are linked to their final income. As mentioned above, only one participant expressed her doubt about the authenticity of the game. However, whether or not this participant was included in the analysis, we fully replicated all findings (refer to the method used in FeldmanHall et al. (2018)). The results section reported the analysis excluding this participant’s data.

## 3. Results

Three-factor repeated measures ANOVA was performed on each dependent variable with facial, vocal attractiveness, and social interest as independent variables by IBM SPSS Statistics 26. Our data are available on the OSF (https://osf.io/s8fyj/) (accessed on 1 February 2025). The descriptive statistics for each condition’s outcome (see Table S1) and the post-test attractiveness ratings both can be found in the Supplementary File. All results of the ANOVA are presented as Table 1.

### 3.1. TG1: Initial Investment Rate

**Figure 2:**
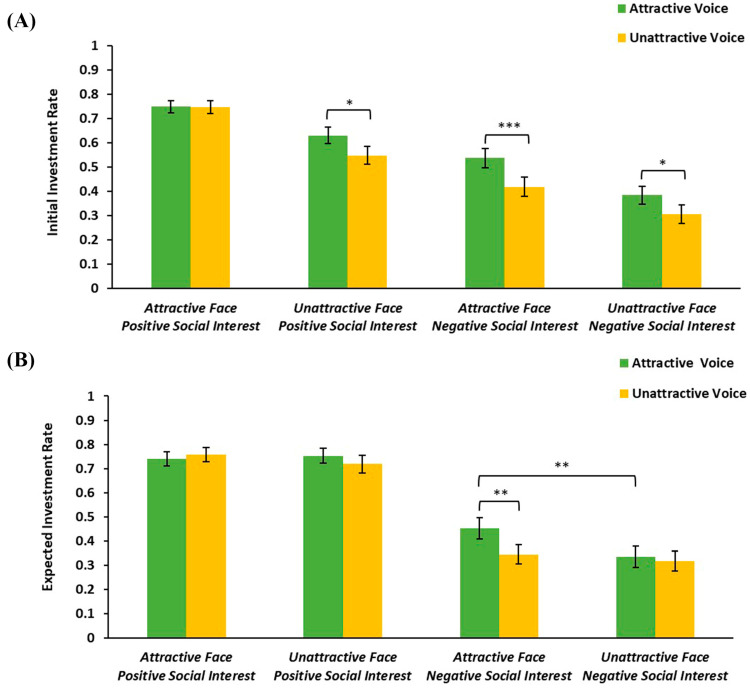
(**A**) Mean initial investment rates as a function of facial attractiveness, vocal attractiveness, and social interest in TG1. (**B**) Mean expected investment rates as a function of facial attractiveness, vocal attractiveness, and social interest in TG2. The error bars represent standard errors. * *p* < 0.05, ** *p* < 0.01, and *** *p* < 0.001.

Participants were more likely to invest when trustees had attractive faces (*M* = 0.61, *SD* = 0.02) than unattractive faces (*M* = 0.47, *SD* = 0.02), *F*(1, 56) = 36.68, *p* < 0.001, *η_p_*^2^ = 0.40, and when trustees had attractive voices (*M* = 0.58, *SD* = 0.02) than unattractive voices (*M* = 0.51, *SD* = 0.02), *F*(1, 56) = 19.77, *p* < 0.001, *η_p_*^2^ = 0.26, also when trustees expressed positive social interest (*M* = 0.67, *SD* = 0.02) than negative social interest (*M* = 0.41, *SD* = 0.03), *F*(1, 56) = 40.72, *p* < 0.001, *η_p_*^2^ = 0.42. The three-way interaction between facial attractiveness, vocal attractiveness, and social interest was significant (Figure 2A), *F*(1, 56) = 4.16, *p* = 0.046, *η_p_*^2^ = 0.07. A simple effect analysis revealed that under attractive-face conditions, when trustees expressed negative interest, participants were more willing to invest in attractive-voice trustees than unattractive-voice trustees, *F*(1, 56) = 14.04, *p* < 0.001, *η_p_*^2^ = 0.20; when trustees expressed positive interest, the difference between attractive and unattractive voices was not significant, *F*(1, 56) = 0.02, *p* = 0.903, *η_p_*^2^ < 0.01. Under unattractive-face conditions, participants’ initial investment rates for attractive voices were higher than for unattractive voices, no matter whether trustees expressed positive social interest, *F*s ≥ 4.95, *p*s ≤ 0.030. Simple effects regarding facial attractiveness and social interest were similar to the main effects described above, *F*s ≥ 7.96, *p*s ≤ 0.007. Other interactions were not significant, *F*s ≤ 3.24, *p*s ≥ 0.077.

### 3.2. TG1: Reinvestment Rate

Separate analyses were conducted on gain trials and loss trials when the participants made investments. On gains when trustees provided repayment, the main effects of facial attractiveness, vocal attractiveness, and social interest in reinvestment rates were all significant, *F*s ≥ 6.04, *p*s ≤ 0.017, indicating that participants were more likely to reinvest in trustees with either attractive faces, attractive voices, or positive interest. On losses when trustees withheld repayment, participants were more willing to reinvest in trustees with attractive faces or voices, *F*s ≥ 5.62, *p*s ≤ 0.021. Other main effects or interactions were not significant, *F*s ≤ 3.45, *p*s ≥ 0.069.

### 3.3. TG2: Expected Investment Rate

Participants expected that investment rates would be higher for attractive-face trusters (*M* = 0.57, *SD* = 0.02) than unattractive-face trusters (*M* = 0.53, *SD* = 0.02), *F*(1, 56) = 6.19, *p* = 0.016, *η_p_*^2^ = 0.10, and higher for positive social interest trusters (*M* = 0.74, *SD* = 0.03) than negative social interest trusters (*M* = 0.36, *SD* = 0.04), *F*(1, 56) = 61.24, *p* < 0.001, *η_p_*^2^ = 0.52. The interaction between social interest and vocal attractiveness was significant, *F*(1, 56) = 6.13, *p* = 0.016, *η_p_*^2^ = 0.10. Importantly, the three-way interaction between facial attractiveness, vocal attractiveness, and social interest was also significant (Figure 2B), *F*(1, 56) = 6.94, *p* = 0.011, *η_p_*^2^ = 0.11. A simple effect analysis revealed that when attractive-voice trusters expressed negative social interest, participants expected that trusters with attractive faces would be more generous than those with unattractive faces, *F*(1, 56) = 12.79, *p* = 0.001, *η_p_*^2^ = 0.19. When attractive-face trusters expressed negative interest, participants expected that trusters with attractive voices would be more generous than those with unattractive voices, *F*(1, 56) = 11.78, *p* = 0.001, *η_p_*^2^ = 0.17. Simple effects of social interest under various conditions were similar to the main effect shown above, *F*s ≥ 25.71, *p*s < 0.001. Other main effects or interactions were not significant, *F*s ≤ 3.50, *p*s ≥ 0.067.

### 3.4. TG2: Reciprocation Rate

When earning investment, participants were more likely to reciprocate trusters expressing positive social interest (*M* = 0.64, *SD* = 0.05) rather than those with negative interest (*M* = 0.41, *SD* = 0.04), *F*(1, 56) = 25.71, *p* < 0.001, *η_p_*^2^ = 0.32. Other main effects or interactions were not significant, *F*s ≤ 3.41, *p*s ≥ 0.070.

## 4. Discussion

Our findings underscore the significant premium effects of beauty and positive social interest in sequential trust decisions involving feedback. However, these effects depend on whether receiving feedback and vary across participants’ roles.

Firstly, attractive faces guarantee better treatment in TG1. No matter whether they receive feedback, participants tend to invest in trustees with attractive faces. Even when attractive trustees did not repay, participants were more willing to reinvest in them. These findings replicated the beauty premium in prior studies (Chen et al., 2012; Voit et al., 2021). Participants would like to trust attractive counterparts even though they withheld repayment, suggesting that beautiful faces may enhance participants’ perception of trustworthiness (Shang & Li, 2020), then increase investments. Pandey and Zayas (2021) also found that when partners with attractive faces incurred financial losses, participants were quicker to forgive them and cooperated with them again in subsequent decisions, which was consistent with our results. In addition, there was a conditional beauty premium for trusters in TG2, such that participants had higher expectations of investment for attractive faces than unattractive faces only when the trusters had attractive voices with negative social interest. When participants perceive their counterparts based on the combinations of stimuli, they may not need to take all factors into consideration since the cognitive resources are limited (Shang & Zhang, 2024b). If the trusters already have attractive voices with positive interest, participants may form a ceiling positive impression and would not consider facial attractiveness. Nonetheless, beauty premiums disappeared when participants received investment: they did not return more money to attractive-face trusters. Ma et al. (2022) stated that role dynamics in economic bargaining leads to a “greater” beauty premium for recipients compared to proposers in the ultimatum game. Similarly, participants would be more cautious about whether to invest since the trustee may keep the whole money. The truster’s decision may be more complex, resulting in preferences for attractive-faced trustees. However, when participants were trustees, although they expected investment, they had no fear after they received the investment since they had the right to withhold the money. Thus, the beauty premium of faces was weaker when participants were trustees rather than trusters.

Secondly, our findings suggested a beauty premium of vocal attractiveness (Shang & Liu, 2022a, 2022b), and it depends on participants’ roles and the type of feedback. When acting as trusters and receiving no feedback, participants were more likely to send money to trustees with attractive voices than those with unattractive voices in most conditions, except when trustees had attractive faces and expressed positive social interest. One possible explanation is that when both attractive faces and positive social interest convey positive signals, which may cause a ceiling effect, participants may pay less attention to vocal attractiveness. However, in the other combinations with negative signals, participants needed to consider vocal attractiveness as a supplement, thus reflecting the “beauty premium” effect. After they knew the feedback, participants would still trust counterparts with attractive voices. What’s more, like the effects of facial attractiveness, the beauty premium of voices only exists in certain conditions when participants were trustees. Only when the trusters had attractive faces and negative social interest, participants expected trusters with attractive voices to pass more money, compared with unattractive-voice trusters, whereas participants did not repay more to attractive-voice trusters. In summary, the beauty premium of attractive faces and voices varied across different combinations of facial attractiveness and social interest, which is similar to Shang and Zhang’s (2024a).

Thirdly, we observed a premium of positive social interest (Shang & Zhang, 2024a; Yuan et al., 2024) which was unaffected by facial or vocal attractiveness. Positive social interest ensured better treatment in decisions except for the reinvestment after loss. The possible explanation is that positive social interest serves as an explicit signal for conveying potential trust cues (Clark, 2008), which was conflict with the financial losses. Participants’ trust in positive social interest was dashed by the loss, eliminating the premium.

Overall, what may happen in the mind of the participant when they perceive and interpret these combinations of stimuli in trust decisions? It is possible that participants integrate stimuli from both the visual and auditory modalities to form an overall impression of the counterpart, which further affects their trust decisions. Wells et al. (2013) and Rezlescu et al. (2015) found that both the attractiveness of targets’ faces and voices influence the overall evaluations of attractiveness, while faces contributed proportionally more than voices. Additionally, when the attractiveness levels of the visual and auditory modalities do not match, people tend to form a relatively negative impression due to disappointment with the negative attractive modality (Zuckerman & Sinicropi, 2011). Furthermore, as the only explicit cue, positive interest generally enhances investments and expectations compared with negative interest, regardless of the other two factors. However, facial and vocal attractiveness also matter even if the partner expresses a negative interest. That is, when participants received negative social signals, they tended to focus more on external visual or auditory attractiveness as a coping strategy to reduce social uncertainty and make relatively confident decisions. As the “what is beautiful is good” stereotype (Dion et al., 1972; Zuckerman & Driver, 1989), participants may not be entirely disappointed with counterparts when their partner’s attitude is negative, and attractive partners receive better expectations than unattractive partners.

There are several limitations in the current research. First, the manipulation of social interest relying solely on voice semantics without genuine social interaction was too artificial, and feedback was also predetermined by researchers, which may limit the generalizability of results. In real social interactions, people probably will not explicitly tell the counterpart their attitudes (even when they really have a positive or negative interest toward him/her). Future studies should adopt more natural methods to manipulate social interest to improve experimental design. Second, this study was within-subject. The repeated use of stimuli across different tasks and within the same task may also introduce some carry-over effects, although we strived to mitigate these influences through counterbalanced design between participants. Also, given we did not record participants’ faces and voices, this may limit how real the trust game could seem to them, although we strived to tell all participants that the game was real. Future research can examine our findings in real scenarios with real persons. Third, the use of single-gender participants and stimuli constrains the broader applicability of the study and prevents the exploration of potential gender differences. Future research should examine the gender difference to develop a more comprehensive research framework. Fourth, a post-test of attractiveness was conducted to verify whether the ratings of stimuli in the formal experiment were consistent with the ratings from the pre-test, serving as a manipulation check of stimuli selection. However, the attractiveness ratings being given after the experiment may be affected by the interactions participants had in the formal experiment. Lastly, in TG2, we did not allow participants to freely choose their expectations and only provided two options: to expect investment or not. If participants did not receive investment, there are no subsequent decisions. We do not know whether a “beauty penalty” exists if investments from attractive counterparts are disappointing.

## 5. Conclusions

In the present trust game, positive social interest is a benefit, except when trustees withheld repayment. Trustees induced a stronger beauty premium for attractive faces and voices than trusters.

## Figures and Tables

**Table 1:** Results of ANOVA for the rates of investment and reinvestment in TG1, the rates of expected investment and reciprocation in TG2 (*N* = 57). The bold values mean *p* ≤ 0.05.

	Factors	*F*	*p*	*η_p_* ^2^
TG1: Initial Investment	Facial Attractiveness	**36.68**	**<0.001**	**0.40**
	Vocal Attractiveness	**19.77**	**<0.001**	**0.26**
	Social Interest	**40.72**	**<0.001**	**0.42**
	Facial Attractiveness × Vocal Attractiveness	0.35	0.555	<0.01
	Facial Attractiveness × Social Interest	0.44	0.512	<0.01
	Vocal Attractiveness × Social Interest	3.24	0.077	0.06
	Facial Attractiveness × Vocal Attractiveness × Social Interest	**4.16**	**0.046**	**0.07**
TG1: Reinvestment (gain)	Facial Attractiveness	**18.39**	**<0.001**	**0.25**
	Vocal Attractiveness	**6.04**	**0.017**	**0.10**
	Social Interest	**31.94**	**<0.001**	**0.36**
	Facial Attractiveness × Vocal Attractiveness	0.02	0.878	<0.01
	Facial Attractiveness × Social Interest	0.03	0.869	<0.01
	Vocal Attractiveness × Social Interest	3.39	0.071	0.06
	Facial Attractiveness × Vocal Attractiveness × Social Interest	1.51	0.224	0.03
TG1: Reinvestment (loss)	Facial Attractiveness	**9.91**	**0.003**	**0.15**
	Vocal Attractiveness	**5.62**	**0.021**	**0.09**
	Social Interest	0.12	0.729	<0.01
	Facial Attractiveness × Vocal Attractiveness	3.45	0.069	0.06
	Facial Attractiveness × Social Interest	0.65	0.424	0.01
	Vocal Attractiveness × Social Interest	1.02	0.317	0.02
	Facial Attractiveness × Vocal Attractiveness × Social Interest	2.56	0.115	0.04
TG2: Expected Investment	Facial Attractiveness	**6.19**	**0.016**	**0.10**
	Vocal Attractiveness	3.32	0.074	0.06
	Social Interest	**61.24**	**<0.001**	**0.52**
	Facial Attractiveness × Vocal Attractiveness	0.63	0.430	0.01
	Facial Attractiveness × Social Interest	3.50	0.067	0.06
	Vocal Attractiveness × Social Interest	**6.13**	**0.016**	**0.10**
	Facial Attractiveness × Vocal Attractiveness × Social Interest	**6.94**	**0.011**	**0.11**
TG2: Reciprocation	Facial Attractiveness	2.97	0.090	0.05
	Vocal Attractiveness	3.41	0.070	0.06
	Social Interest	**25.71**	**<0.001**	**0.32**
	Facial Attractiveness × Vocal Attractiveness	0.65	0.424	0.01
	Facial Attractiveness × Social Interest	0.08	0.785	<0.01
	Vocal Attractiveness × Social Interest	1.60	0.212	0.03
	Facial Attractiveness × Vocal Attractiveness× Social Interest	0.97	0.328	0.02

## Data Availability

The data supporting the conclusions of this article are available on OSF (https://osf.io/s8fyj/) (accessed on 1 February 2025) or upon reasonable request from the authors.

## Supplementary Materials

The following supporting information can be downloaded at https://www.mdpi.com/article/10.3390/bs15020175/s1: Table S1: Means and standard deviations for the outcomes of TG1 and TG2 (*M* ± *SD*).
