# Supplementary material for: Beauty Is Not Always a Perk: The Role of Attractiveness and Social Interest in Trust Decisions
Source: Behav Sci (Basel). 2025 Feb 7;15(2):175. doi: 10.3390/bs15020175 (PMC11851420; doi:10.3390/bs15020175)
Supplement: Supplementary file 1 [file behavsci-15-00175-s001.zip › behavsci-3291115-supplementary.pdf]

Table S1. Means and standard deviations for the outcomes of TG1 and TG2 (*M*±*SD*).

		AAP	AAN	AUP	AUN	UAP	UAN	UUP	UUN
TG1	Initial Investment	0.75 (0.18)	0.54 (0.30)	0.75 (0.20)	0.42 (0.30)	0.63 (0.26)	0.38 (0.28)	0.55 (0.28)	0.31 (0.30)
	Reinvestment (gain)	0.67 (0.28)	0.48 (0.33)	0.68 (0.32)	0.36 (0.35)	0.55 (0.31)	0.32 (0.33)	0.52 (0.34)	0.26 (0.29)
	Reinvestment (loss)	0.08 (0.13)	0.11 (0.18)	0.10 (0.22)	0.07 (0.16)	0.08 (0.16)	0.06 (0.16)	0.02 (0.09)	0.02 (0.09)
TG2	Expected Investment	0.74 (0.23)	0.45 (0.34)	0.76 (0.22)	0.35 (0.30)	0.75 (0.23)	0.34 (0.32)	0.72 (0.28)	0.32 (0.31)
	Reciprocation	0.64 (0.39)	0.46 (0.35)	0.66 (0.41)	0.40 (0.36)	0.64 (0.37)	0.42 (0.35)	0.60 (0.36)	0.36 (0.38)

Notes: AAP = attractive faces, attractive voices and positive social interest. AAN = attractive faces, attractive voices and negative social interest. AUP = attractive faces, unattractive voices and positive social interest. AUN = attractive faces, unattractive voices and negative social interest. UAP = unattractive faces, attractive voices and positive social interest. UAN = unattractive faces, attractive voices and negative social interest. UUP = unattractive faces, unattractive voices and positive social interest. UUN = unattractive faces, unattractive voices and negative social interest.

Post-Experiment Attractiveness Ratings Results

We used an independent samples *t*-test on the mean facial attractiveness ratings. Results showed that attractive faces (M = 6.11, SD = 0.46) were perceived as more attractive than unattractive faces (M = 3.13, SD = 0.50), $t(14) = 12.36$, $p < 0.001$, 95% CI = [2.46, 3.49]. Besides, a 2 (vocal attractiveness: attractive; unattractive) × 2 (social interest: positive; negative) ANOVA on the mean vocal attractiveness ratings revealed a significant main effect of vocal attractiveness, $F(1,12) = 247.02$, $p < 0.001$, $\eta_p^2 = 0.95$, where attractive voices (M = 6.47, SD = 0.11) were rated as more attractive than unattractive voices (M = 3.94, SD = 0.11). The main effect of social interest or the interaction between social interest and vocal attractiveness were both not significant, Fs ≤ 0.01, ps ≥ 0.879. These results suggested that experimental stimuli were effective.
